# Supplementary material for: A need or a luxury? Parents’ attitudes towards international schools from a linguistic perspective
Source: PLoS One. 2026 Apr 24;21(4):e0314671. doi: 10.1371/journal.pone.0314671 (PMC13108773; doi:10.1371/journal.pone.0314671)
Supplement: S1 Appendix — (DOCX) [file pone.0314671.s001.docx]

Appendix 1 (Questionnaire)

**Parents' choice of international schools or programs for their children**

This questionnaire aims to examine the opinions of parents of male and female students

regarding choosing international schools or programs for their sons and daughters.

An international program is a program in which all courses are taught in a language other than Arabic (usually English).

This study is intended for use for scientific research purposes, and the information included in it will remain confidential and not subject to publication outside the research framework.

By moving to the next pages and completing this questionnaire, the participant agrees to be part of the study.

We thank you for your cooperation and publishing the questionnaire

For any inquiries, you can contact the researchers via email [2023SSRG@gmail.com](mailto:2023SSRG@gmail.com)

* Indicates required question

1. Legal capacity *

*Mark only one oval.*


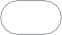
 Father
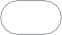
 Mother
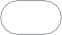
 Other

1. Age *

*Mark only one oval.*


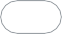
 20-26


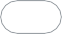
 27-33


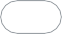
 34-40


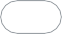
 41-47


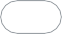
 48-54


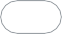
 55-61


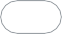
 62 and above

1. Educational qualification *

*Mark only one oval.*


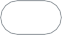
 Intermediate school
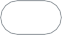
 Secondary school
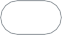
 Bachelore


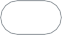
 MA


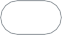
 PhD

1. Martial status *

*Mark only one oval.*


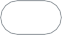
 Married
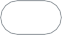
 Divorced


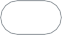
 Another guardian
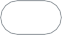
 Widow

1. Both parents monthly income. *

*Mark only one oval.*


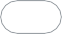
 5000-12000


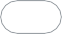
 13000-20000


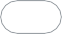
 21000-28000


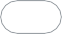
 29000-36000


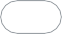
 36000-42000


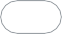
 42000-48000


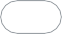
 49000 and above

1. Do you have sons/daughters who are currently studying or have previously *

studied in international schools or programs?

*Mark only one oval.*


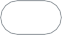
 Yes and they are still studuing there. *Skip to question 7*


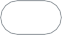
 They were in interanational programs but have now moved to other internationsl schools *Skip to question 7*


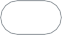
 They were in international schools but have now moved to Arabic schools.

*Skip to question 7*


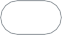
 They continued in international programs until graduation. *Skip to question 7*


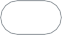
 I have no children studying in international schools

Parents of students in international schools or programs

1. Do all of your children study in international schools? *

*Mark only one oval.*


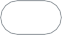
 All of my children are studying in international schools
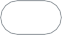
 Some of my children study in international schools

1. Assuming that you are financially capable, will you choose to have all your *

children study in international programs?

1. If some of your children are in international schools...what is the reason for *

others to enroll in other programs/schools?

1. Have you ever been enrolled in a sponsorship overseas, or spend more than a *

year overseas on duty?

*Mark only one oval.*


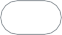
 Yes, i was enrolled in a a sponsorship.
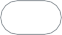
 Yes, i was on a duty overseas.


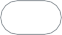
 Non of the options above is applicable.

1. If you have had a previous experience of scholarship or expatriation, have *

some or all of your children attended schools abroad?

*Mark only one oval.*


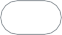
 Yes, some or all of my children have attended nurseries, kindergartens or schools


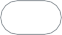
 I was sent on scholarships previously, but my children did not attend any nurseries/kindergartens/schools


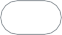
 I didn't have children then


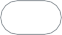
 Not applicable (I have never been on scholarship or expatriation)

1. If you have children who are currently studying or have previously studied in *

international programs, what are the reasons for choosing this program? Please detail the answer

1. Which of the following skills do you want your children to master in international *

programs?

*Mark only one oval.*

The linguistic part (English only) The scientific part only.

Both language and other sciences.

Other:

1. How do you describe the level of your son/daughter studying in international *

programs in the Arabic language?

*Mark only one oval.*


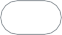
 Excellent


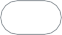
 Above average
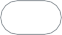
 Average


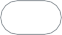
 Weak
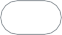
 Very weak

1. How do you describe the level of your son/daughter studying in international *

programs in the English language?

*Mark only one oval.*


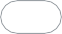
 Excellent


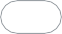
 Above average
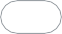
 Average


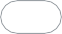
 Weak
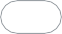
 Very weak

1. Which languages do you think are most important for a student’s educational *

and career future?

*Mark only one oval.*


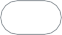
 Arabic
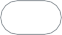
 English

1. I chose international programs because of my knowledge of previous models *

who excelled academically or professionally after graduating from this program

*Mark only one oval.*


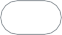
 Strongly agree
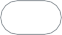
 Agree


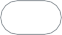
 Neutral
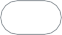
 Disagree


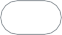
 Strongly disagree

1. I chose international programs because I expected that graduates of * international programs would have a competitive advantage compared to their peers in other programs

*Mark only one oval.*


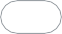
 Strongly agree
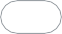
 Agree


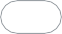
 Neutral
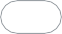
 Disagree


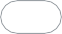
 Strongly disagree

1. I chose international programs because I expected that graduates of *

international programs would have English language abilities that would set them apart from their peers

*Mark only one oval.*


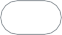
 Strongy agree
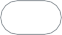
 Agree


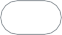
 Neutral
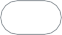
 Disagree


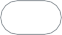
 Strongly disagree

1. I chose international programs because I expected that graduates of * international programs would have academic abilities that would set them apart from their peers

*Mark only one oval.*


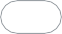
 Strongly agree
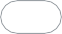
 Agree


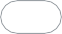
 Neutral
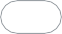
 Disagree


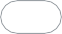
 Strongly disagree

1. I chose international programs because of the excellence of the services *

provided to the students of those programs in terms of the quality of the buildings and good treatment

*Mark only one oval.*


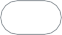
 Strongly agree
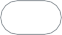
 Agree


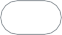
 Neutral
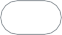
 Disagree


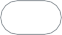
 Strongly disagree

1. I chose the international programs because of their strong reputation *

*Mark only one oval.*


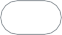
 Strongly agree
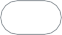
 Agree


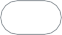
 Neutral
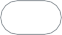
 Disagree


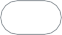
 Strongly disagree

1. Because their financial cost is high, the outcomes of international programs will *

often be distinguished

*Mark only one oval.*


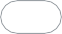
 Strongly agree
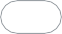
 Agree


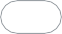
 Neutral
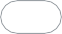
 Disagree


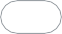
 Strongly disagree

1. I make sure that my son studies in international programs because the student *

environment is often distinguished

*Mark only one oval.*


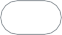
 Strongly agree
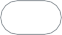
 Agree


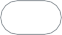
 Neutral
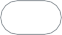
 Disagree


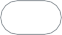
 Strongly disagree

1. I am keen for my son to study in international programs because they adopt *

curricula from scientifically advanced countries

*Mark only one oval.*


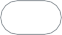
 Strongly agree
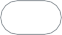
 Agree


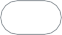
 Neutral
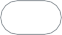
 Disagree


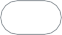
 Strongly disagree

1. My children, or some of them, study in international schools because they do * not master Arabic and it is difficult for them to adapt to the national programs offered in Arabic.

*Mark only one oval.*


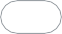
 Strongly agree
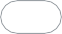
 Agree


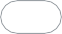
 Neutral Disagree

Strongly disagree

1. I feel special for my son/daughter, as they are able to study in international *

programs

*Mark only one oval.*

Strongly agree Agree

Neutral Disagree

Strongly disagree

1. I chose international programs because I expected that graduates of * international programs would have better chances of being accepted into universities than others

*Mark only one oval.*

Strongly agree Agree

Neutral Disagree

Strongly disagree

1. Studying in international schools negatively affects the student’s identity and *

integration into society

*Mark only one oval.*

Strongly agree Agree

Neutral Disagree

Strongly disagree

1. Do you have anything to add about this?
2. If you are interested in this topic and do not mind conducting a short interview about it, please include your mobile number or email.
