# Supplementary material for: A need or a luxury? Parents’ attitudes towards international schools from a linguistic perspective
Source: PLoS One. 2026 Apr 24;21(4):e0314671. doi: 10.1371/journal.pone.0314671 (PMC13108773; doi:10.1371/journal.pone.0314671)
Supplement: Attachment — Submitted filename: Response to the editor and reviewers.docx [file pone.0314671.s002.docx]

Response to the editor and reviewers

Dear editor

Thanks for your valued comments. The manuscript has now been revised, taking editor’ comments into account. Two versions are now submitted, the original copy, and a revised copy with tracked changes. Referencing style in the revised version is now according to PLOS ONE referencing style. Also, the ethics part was revised to clarify the ethical procedure during data collection. Also, a copy of the questionnaire is uploaded.

Thanks for your patience and looking to hear from you soon.
