# Supplementary material for: A need or a luxury? Parents’ attitudes towards international schools from a linguistic perspective
Source: PLoS One. 2026 Apr 24;21(4):e0314671. doi: 10.1371/journal.pone.0314671 (PMC13108773; doi:10.1371/journal.pone.0314671)
Supplement: Attachment — Submitted filename: Response to Reviewers.docx [file pone.0314671.s003.docx]

Additional Editor Comments (if provided):

The manuscript presents a relevant and potentially valuable topic; however, it requires substantial revision to meet publication standards. The research questions lack depth and insight, and the study’s novelty and distinction from existing literature are not clearly articulated. The literature review does not sufficiently justify or support the identified research gap, and several sections suffer from semantic redundancy due to repetitive phrasing. The conclusion reads more like a list than a cohesive synthesis, lacking logical connections between ideas. The sample size is too limited to generalize the findings, and methodological details—including sampling regions, inclusion criteria, data collection process, and survey validation—are inadequately described. The absence of the questionnaire in the appendix further weakens the study’s transparency. Moreover, the discussion does not critically engage with the findings, omits study limitations, and fails to offer practical implications or future research directions. The writing requires major language refinement to improve clarity, grammatical consistency, and coherence, with particular attention to verb tenses, pronoun references, and sentence transitions. The introduction and certain subsections need to be more concise, incorporating recent developments such as the growing influence of social media on education in Saudi Arabia. Overall, the paper must undergo major revision to enhance its theoretical contribution, methodological rigor, linguistic quality, and overall scholarly value.

Dear Editor 1

Thanks for your insightful and valuable feedback. The researchers have worked on revising the whole manuscript for more than a month. Editor and reviewers’ comments were tracked and worked on one by one. To enhance the quality of the language, the manuscript was then proofread by a quality proofreader Scribendi ([www.scribendi.com](http://www.scribendi.com)). I have attached a copy with tracked changes as well as clear version of the manuscript.

Thanks again for your insightful comments

Reviewers' comments:

6. Review Comments to the Author

Reviewer #1: 1. The abstract of the paper is written well.
2. The statistical analysis and the illustrations are adequately done.
3. However, research questions framed are not so insightful.

Research questions are now rewritten to be narrowed down and more straightforward
4. The study's novelty is not mentioned. How does the study differ from the existing one?

We added few sentences in the introduction (just before the research questions) clarifying the novelty of this research.
5. The literature review section does not say anything in support of the research gap identified and addressed.

The last 2 sentences in section 2.2 in the literature review was added to clarify and elaborate on the research gap.

6. Semantic redundancy is created by the repetitive use of certain phrases "global perspectives," "interconnected world," "multicultural society," "cultural exchange" "Significant role" and "pivotal role." This needs to be addressed by the researchers.

These phrases were revised and edited in the entire manuscript.

7. The concluding statement of the paper says --- “global communication, preparation for higher education, enhanced career opportunities, the facilitation of mobility and cultural exchange” — provide a significant advantage for English as a medium of instruction. It reads more like a bullet point list than an integrated sentence. The logical links between English and each of these points are asserted but not explained.

Revised and edited to be linked only to the findings of the study.

8. The sample of the study was very small (quantitative and qualitative data through a 30-item questionnaire to which 76 parents responded) and the authors try to claim to address the needs of the students of Saudi Arabia.

The researchers tried to reach as much number of participants as possible for several weeks, but only 76 parents agreed to participate. This has also been mentioned in the limitations of the study.

9. Instead of providing one-side view, the authors can acknowledge challenges and try to provide possible ways to resolve it.

More argumentative points raised to raise questions to be solved.

10. The authors can avoid using vague transitions in sentences. Try to use bridging sentences.

The structure of many sentences and paragraphs was rewritten/reorganized/rephrased to avoid vagueness.

11. The authors ought to eliminate generalized claims and think of adding specific examples.

Detailed examples are now provided, focusing on the Saudi contexts

12. The authors need to remove the unsubstantiated references found in support of their argument. Proper citation is the way out.

More UpToDate relevant references were added to the manuscript.

13. The Questionnaire used during the pre-study period was not provided in the appendix.

A copy of the questionnaire is now in the appendix.

14. The standard of English needs to be improved.

The entire manuscript is now edited using a quality proofreader Scribendi ([www.scribendi.com](http://www.scribendi.com)).

15. Grammatical Errors need to be addressed:
Verb Tense Consistency:
• “Another major reason for enrolling children in international schools is if they were born or raised abroad…”
→ “is if they were” → awkward and inconsistent; consider parallel tense.
Incorrect or vague pronoun reference:
• “These are important for the future of the children.”
→ “These” refers to “quality of the curricula and the environment” but is too vague here.
Lack of Clarity in the Final Sentence found in the Discussion:
• “This could affect their perceived status and prestige, as well as the credibility of their educational outcomes.”
→ Ambiguous: whose perceived status — the schools or the students?

Grammatical check has been done. In addition, the entire manuscript is now edited using a quality proofreader (Scribendi) ([www.scribendi.com](http://www.scribendi.com)).

16. Scope for further is research is not given.

Scope for further research is now included in the conclusion

17. Hence it is perceived that the study lacks many important and crucial elements in support of the claims made by the authors.

Researchers elaborated more to strengthen the claims in the study

18. Therefore, the paper needs to be sent for major revision.

The paper has now been fully revised by the authors.

19. The following article can be cited in the revised manuscript as there are some relevant ideas in support main argument of the research paper in the section: 2.3 Impact on Human Skills and Education:
Asokan Vasudevan, K. T. Tamilmani, Suleiman Ibrahim Mohammad, V. S. Sridheepika, N. Raja, Eddie Eu Hui Soon, Sriganeshvarun Nagaraj, and Ahmad Samed Al-Adwan. (2025). Voices of Concern: Contemplations on Socioeconomic, Ethical, and Developmental Impacts of AI. Applied Mathematics and Information Sciences: An International Journal, 19 No.1, 221-232. ISSN 2325-0399 (online) <https://ddei5-0-ctp.trendmicro.com:443/wis/clicktime/v1/query?url=https%3a%2f%2fwww.naturalspublishing.com%2fArticle.asp%3fArtcID%3d29328&umid=95BD2C90-412B-C206-81FF-9C007C43FFDB&auth=0f471a1006833f12759dea0d050a257e25d54c39-a1bb048fa8223827de42900b51a34b55c782af67>

Thanks for the recommendation. Very useful article.

Dear Reviwer 2: Thanks for your insightful and helpful comments. The authors have now made major revision taking all of your comments into considerations, one by one.

Reviewer #2: - The topic is interesting, but the study itself does not add much to the reader’s knowledge and does not provide future directions to make the results valuable

Scope for further research is now included in the conclusion

-Language used in the article should be revised for better choice of words and punctuation. -The title implies more outcomes than the paper provides.

The entire manuscript is now edited using a quality proofreader (Scribendi) ([www.scribendi.com](http://www.scribendi.com)).

Introduction:
The introduction provides valuable information to help readers understand the educational system in Saudi Arabia; however, it would be more helpful if it were more concise.
The studies on “Saudi Arabians’ attitudes towards English” are outdated and may not accurately reflect the current status in Saudi Arabia, given the significant changes in the country and the shift toward extensive social media use over the last few years. This change should be reflected as well, and might add a great understanding to the topic

The researchers have elaborated more and mentioned these recent changes in details.

2.4. Classrooms: It would be helpful to provide the reader with the actual number of students in public vs private schools in Saudi Arabia to be able to decide on the difference and to understand if the class size is appropriate or not. In addition, whether this trend of choosing private schools over public ones has increased over time. Again, it would help the reader to have this part written in a summarized, concise way instead of mentioning different studies with different results

The number of students is now clarified

Sample: Which regions of Saudi Arabia were included in the study? What were the inclusion and exclusion criteria? The specific results on the sample should be added under
the results section.

It is not clear from which regions do participants come from. However, the authors who distributed the questionnaire live in 4 regions of Saudi Arabia.

Data collection:
The data collection section should be revised to provide a clear description of the process. It is mentioned that “The questionnaire was then distributed to various individuals at universities and other educational institutions. We also asked the participants to distribute it to others through a snowballing process to enhance the quality of the study.” Why were individuals at universities directly targeted, and wouldn't this create bias? And what is meant by educational institutions? Do you mean schools?

-Yes, changed to schools instead of educational institutions.

-Distributing the questionnaire started from the authors who work in Universities, but the questionnaire was actually sent to participants who work in different domains (some of the parents do not even work -as they mention in the results of the questionnaire.

This section has information regarding developing the survey. It is recommended to separate the information into another section. Regarding the survey, it needs to be described more straightforwardly; it is not clear what the survey questions were. Perhaps it would be helpful to include them. More importantly, the survey’s validity was not mentioned, and no piloting was done.

The questionnaire is now added in the appendix.

The questionnaire has also been validated by an academic who are specialists in the field.

Findings:
This section is difficult to follow; it would benefit from being more focused to aid the reader's understanding.
In sections 4.1 and 4.2, the percentages of parents providing information are not included, which does not accurately represent the value of the results.

Thanks for this suggestion. I agree. Many of the findings are now reorganized to be easy to follow by the reader.

Discussion:
The results of this study are not surprising and may not add to our knowledge unless the authors apply their results to our reality. For example, you use the results to provide suggestions to improve the educational system.
Limitations of the study are not mentioned

Limitations and scope for future research are now added in the conclusion.
