# Supplementary material for: A need or a luxury? Parents’ attitudes towards international schools from a linguistic perspective
Source: PLoS One. 2026 Apr 24;21(4):e0314671. doi: 10.1371/journal.pone.0314671 (PMC13108773; doi:10.1371/journal.pone.0314671)
Supplement: Attachment — Submitted filename: Response_to_reviewers_auresp_3.docx [file pone.0314671.s004.docx]

Response to reviews

|  | Reviewer comment | Response |
| --- | --- | --- |
| 1 | In abstract, a mixed-methods research design was employed; however, this is not explicitly mentioned within the abstract. Additionally, numerical values should be incorporated to substantiate the study’s findings. In addition, a survey for quantitative data collection is mentioned, the procedures for qualitative data collection are not specified. | Research design was addressed in the abstract. The numerical values from the quantitative finding section were also added. Both quantitative and qualitative data were collected from the questionnaire. We rephrased the data collection procedure statement to make it clearer. |
| 2 | The literature review is clearly articulated and supported by adequate references; however, recent studies (2025, 2024, 2023, and 2022) should be incorporated to strengthen the relevance. | Recent studies (2022-2025) were added to strengthen the relevance |
| 3 | In the methodology section, the demographic details should be corrected as 52 male respondents (68.4%) and 24 female respondents (31.6%). Furthermore, no explanation is provided regarding the qualitative component of the study, which weakens the mixed-methods research method. Furthermore, mention number of demographic details and Likert scale questions separately. | The demographic details were corrected in 3.2  Further explanations and elaborations on the qualitative analysis are added  -Number of demographic details questions are added  -Likert-scale is now explained (the five options) |
| 4 | In the findings section, the quantitative results are clearly presented and interpreted. However, the qualitative findings are not addressed. It is recommended that qualitative results be presented under a separate subheading with proper thematic analysis. Correlate the Likert scale questions and open ended questions to the research questions with proper justification. | Emerged themes were linked to research questions as requested. |
| 5 | In the discussion and conclusion sections, the research questions are not sufficiently justified in relation to the findings. These sections should be revised and justified to each research question. | Each research question was highlighted and included in the discussion section with related answers. Research questions were not listed in the conclusion due to the nature of this section to be a summary with less details. |
